# Chronic leaf harvesting reduces reproductive success of a tropical dry forest palm in northern Mexico

**DOI:** 10.1371/journal.pone.0205178

**Published:** 2018-10-18

**Authors:** Leonel Lopez-Toledo, Angeles Perez-Decelis, Franceli Macedo-Santana, Eduardo Cuevas, Bryan A. Endress

**Affiliations:** 1 Instituto de Investigaciones sobre los Recursos Naturales-Universidad Michoacana de San Nicolás de Hidalgo, Morelia, Michoacán, Mexico; 2 Facultad de Biología-Universidad Michoacana de San Nicolás de Hidalgo, Morelia, Michoacán, Mexico; 3 Eastern Oregon Agriculture and Natural Resource Program-Oregon State University, La Grande, Oregon, United States of America; Natural Resources Canada, CANADA

## Abstract

Palm leaves represent one of the most important non-timber forest products in tropical and subtropical regions. *Brahea aculeata* is an endemic palm of northwest Mexico, whose leaves are intensively exploited for roof thatch and handcrafts. As part of a long-term defoliation experiment, we evaluated the effects of different leaf harvest on foliar and reproductive traits of adults and attributes of their progeny. We conducted a six-year manipulative experiment and applied three harvesting treatments to adults: high harvest, low harvest and no harvest (control). We recorded leaf production and size, flower and fruit production, seed germination and seedling growth. We also explored trade-offs among foliar and reproductive traits.

Harvested palms exhibited drastically reduced reproductive activity, producing fewer flowers and fruits (up to 80 and 90% fewer than unharvested palms). However, individuals in both harvest treatments had larger leaves and increased leaf production rates, compared to control palms. For harvested palms, we registered first a slight increase in leaf traits and a decline in reproductive attributes. These traits showed a gradual reduction and for six period attained very low proportional values compared to control palms (~0.10), however individuals in the harvested treatments maintained the greatest leaf lengths and leaf production rates. Seed germination and seedling growth rates of progeny from harvested palms were significantly lower than control palms, with seeds from the high harvest treatment having the lowest seed production and germination rates. Relationships among leaf (size/production) and reproductive traits (flower/fruit production) were positive during the fourth year, but showed negative relationships for the fifth year suggesting a trade-off between reproduction and growth functions. Leaf harvesting in *B*. *aculeata* seems to alter patterns of resource allocation away from reproduction as reflected in a decrease in the probability of reproduction, seed number, germination, and vigor, causing a strong decrease in the reproductive success of this species. Results showed that the consequences of long-term leaf harvest not only affect harvested individuals, but also the fitness and vigor of progeny. This type of long-term studies is essential to understand the population dynamics of non-timber forest products and helps inform sustainable harvesting programs considering intensity, frequencies and periods for recovery from defoliation. Also results may help to explain how intensive and non-planned management schemes may negatively affect vital rates and long-term dynamics of populations from non-timber forest products and other components of the ecosystem.

## Introduction

In the tropics and subtropics, palms are one of the most sources of non-timber forest products (NTFP, [1–4]). Palms provide a wide range of products including leaves, fruits, inflorescences and trunks, that are used for building material (e.g. roofs, floors), food, medicine, the production of fibers and oils, and many other cultural, subsistence or commercial purposes [5], [3], [63]. However, excessive extraction can negatively impact morphological and structural traits of harvested individuals, which can then affect plant vital rates (growth, survival, reproduction) and population dynamics [6–8], [55].

In general, previous studies have shown that many palm species are resilient to defoliation [9,10]. However, effects vary among species, and depend on the intensity and frequency of leaf removal [11]. As a result, a wide range of responses to defoliation have been reported. In physiological terms, responses include positive (over-compensation), negative (under-compensation) or neutral (little or no difference from unharvested palms) [12–14], [16]. Compensatory mechanisms suggest an alteration in the processes related to photosynthesis, the reallocation of resources within individuals, or the mobilization of stored resources in roots, stems or other storage organs to produce new leaves to replace the leaf area lost [14,15], [19]. Thus, based on the principle that resources are limited, assigning them to a single function should have negative consequences for other functions, resulting in trade-offs between vegetative and reproductive traits [16–17], [20, 21].

Because leaves from a wide range of palm species are economically and/or culturally important, a number of studies have explored the effects of harvest on individuals [9, 10], [14]. Studies have been carried out on species found in both wet and dry tropical and subtropical ecosystems, including species such as *Brahea dulcis*, *Sabal mexicana*, *S*. *yapa*, *Chamaedorea radicalis*, *C*. *oblongata and C*. *quezalteca* to name a few [6], [8], [10], [22], [23]. In experiments with a single defoliation event, most studies do not report negative effects on foliar or reproductive traits, while some even report positive effects [11], [24], [55]. In contrast, for palms such as *Astrocaryum mexicanum*, *Neodypsis decary*, *Chamaedorea elegans* and *Chamaedorea ernesti-agustii*, growth, survival and production of flowers and fruits were affected by increasing the frequency of defoliation events [7,8], [25, 26, 27]. While a number of studies have explored the effects of defoliation on palm vital rates and population dynamics, most experiments were short-term (1–2 years). We are not aware of long-term studies that have examined the effects of chronic harvesting on leaf and reproductive traits, on progeny success, and the possible trade-offs between vegetative and reproductive traits that could manifest at high intensities and/or high frequencies of leaf harvest. These questions are important to address and are necessary to expand our knowledge and understanding of the effects of defoliation on palm vital rates, patterns of resource allocation, the effects of defoliation on components of reproductive success and subsequent success of progeny. These are important factors affecting long-term population dynamics of species, but actually are still poorly understood [4], [28].

In this study, we evaluated the effects of chronic defoliation on *Brahea aculeata*, an endemic palm found in tropical dry forests of northwest Mexico [29] through a six-year manipulative experiment. The aim of this study was to evaluate the effects local harvesting schemes on *B*. *aculeata*, and inform sustainable management of the species. Specifically, we investigated the effects of leaf harvest on leaf traits (leaf production and size), reproductive success (production of flowers, fruits and probability of reproduction) and progeny performance (germination and seedling growth). This effort builds on previous research, that found few effects of leaf harvest on foliar and reproductive traits on *B*. *aculeata* populations over three years [22], [30]. After the first three years of the experiment, results indicated that individuals of *B*. *aculeata* were resilient to defoliation and some compensatory responses and resource re-allocation of resources were observed, with increases in leaf production found in harvest treatments, accompanied by slight declines in reproductive output [22], [30].

In this paper, we report the effects of three experimental leaf harvest intensities (none, low and high) on large reproductive individuals (>2.5 m) over six years (2011–2016). We expected palms to be resilient to low intensity management (annual frequency and low harvesting) and as a result of overcompensation process palms will show low effects on leaf and reproductive traits; whereas during the fifth and sixth year of the experiment, individuals subjected to high intensity and increase in frequency of harvest (semiannual and high harvesting) due to reduction in stored reserves would result in declines in leaf traits, but mainly in the reproductive output and vital rates of progeny. In addition, we expected that negative relationships between leaf and reproductive traits over time, indicating trade-offs would become evident as once-stored resources are depleted and resources allocation is mainly for vegetative traits. Given that populations of *B*. *aculeata* seems to be declining, we discuss how intensive harvesting of leaves may contribute to this decline [29]. Finally, we argue that robust and long-term studies regarding the effects of leaf harvest on plant vital rates are essential to inform conservation, management, and sustainable harvest of this and other non-timber forest products.

## Materials and methods

### Study species and its management

*Brahea aculeata* is threat species in Mexican red list and therefore we obtained the permission to experimentally harvest leaves. The General Department of Wildlife from the Agency of Environment and Natural Resources (SEMARNAT) from Mexican Government are responsible to grant the permission (Permit numbers: SGPA/DGVS/01991/10 and SGPA/DGVS/10652/10). *B*. *aculeata* (Brandegee) H. E. Moore, (*Erythea aculeata* Brandegee) is a palm, endemic to Northwestern Mexico, in the states of Sinaloa, Sonora, Chihuahua and Durango. It is a single-stemmed palm 3–8 m in height with palmate leaves and large inflorescences of 100–150 cm in length with hermaphrodite flowers [31–33]. The fruits are slightly globose, with one seed per fruit. It flowers from March to May and the fruits ripen in March of the following year [31]. This palm is listed as ‘vulnerable” on the IUCN Red List and classified as endangered in the Mexican Red List, however no proper studies have been carried out [33–35].

In the area, six palm species are important as a non-timber forest products and *B*. *aculeata* represent the most important [5], [29]. Leaves of this species are used intensively for the manufacture of handicrafts and the construction of roofs for houses and buildings, particularly for the tourist industry on the shore of the Sea of Cortés [22], [29]. Commercial leaf harvesting in the region has been carried out for at least 50 years, with local residents developing traditional strategies for leaf harvest and palm management [5], [29]. In general, there are two principal leaf-harvesting schemes in the area: i) The first, which is conducted by long-term rural residents of the region, hereafter referred as “Low harvest”, and the ii) Second one applied by non-native people, hereafter defined as “High harvest”. The former usually involves harvesting of all but the two-three newest fully expanded leaves and no more than one spear leaf. In contrast, the “High harvest” involves a more aggressive harvesting scheme, cutting all available leaves and spear leaves (leaves yet to expand). Depending on demand for leaves, some areas may be harvested every six months, every year or every two years [29].

### Study area

The research was conducted in the Sierra de Álamos-Rio Cuchujaqui Reserve (RSA-RC) in the state of Sonora. Elevation in the reserve ranges from 300 to 1600 masl, resulting in a gradient of vegetation from tropical dry forest to pine-oak forest [36]. Based on the local meteorological station, annual precipitation is highly variable, and averages 650 mm/year (range: 190–1120 mm). The dry season lasts 8 months (November to June) and it is very pronounced, with just 25–35% of the total annual rainfall occurring during this time. The average annual temperature is 21.5 ° C (range: 10–41 °C, respectively). Our experiment was conducted in the upper basin of the Cuchujaqui river (27º12'30 "- 26º53'09" N and 109º03'00 "- 108º29'32" W) at an elevation of 520–540 masl. This area represents the northernmost tropical dry forests in North America. The canopy reaches to 10 meters and dominant tree species include: *Lysiloma divaricatum*, *L*. *watsonii*, *Ipomoea arborescens*, *Brogniartia alamosana*, *Ceiba acuminata*, *Tabebuia impetiginosa*, *Pseudobombax palmeri*, *Bursera* spp. and *Pachycereus pecten-aboriginum* [33], [36]. In the area, *B*. *aculeata* is distributed within the tropical dry forest zone with large variation in densities ranging from 50 to 300 individuals ≥ 1.0 m height per hectare [29].

### Experimental design

This study represents a continuation of a defoliation experiment that began in the reproductive season 2010–2011 (see [22], [30]). Then, we established six permanent plots of 25 x 25 m at Los Llanos, a private property with the largest density of palms in the area which had not been harvested in the two previous years [22], [29]. We mapped and measured all *Brahea aculeata* stems (seedlings ≥ 5 cm height up to 8 m stems) within each plot (1150 individuals) and harvesting treatments were randomly assigned [30]. All palms used in the experiment were healthy and located in the same stand with similar forest cover and altitude (520–540 m asl). The straight distance among individuals was of no more than 400 m. The density of all size individuals at each plot was very similar with 180–220 individuals/625 m^2^. During the first three years (2011–2014) individuals were subjected to one of three annual harvest treatments. The treatments applied were as those traditionally used in the area and above outlined: 1) High harvest (H) and 2) Low harvest (L) and 3) Control (C), where no leaves were removed. In the first part of the experiment (2011–2014), all size palms (seedlings ≥ 5 cm height up to 8 m stems) were subjected to annual leaf harvesting to explore effects on functional and demographic patterns [30]. In the second part of the study, we selected large reproductive individuals (2.5–3.0 m high) from the original dataset and subjected them to a more intensive management (semiannual frequency with), which is also common at the study area. Thus, in this study we compared the leaf and reproductive traits over six year of study which include measurement before harvesting (2010–2011) and represents the baseline to compare the effects of leaf harvesting treatments, the annual harvest (2011–2014) and the semiannual harvest (2014–2016). Annual harvesting was applied in January, while semiannual harvesting was applied in January and July each year. Semiannual harvesting was applied following the same treatments mentioned before (High harvest, Low harvest and Control). Due to the detail of the present study, we randomly selected only 15 reproductively-active adults per treatment, which leaf and reproductive attributes were monitored in detail. Due to uncontrolled and illegal harvesting over the study period, we lost some individuals, which reduced our sample size from 15 to 7 (C), 10 (L) and 13 (H) and conducted all analysis with these individuals.

#### Leaf traits

To evaluate the effect of the defoliation on foliar traits, leaf production, total number of living leaves and leaf length were recorded for each individual. To quantify leaf production, after each harvest, the newest leaf was marked. Leaf length was measured on the second newest leaf of each individual as this represents a fully developed leaf.

#### Reproductive success

As indicators of reproductive success, we counted the number of flowers, fruits and obtained the probability of reproduction. Flowers were only registered during 2014–2016, while fruits and probability of reproduction were estimated since the before-harvest measurement (2010–2011). In February 2014 and 2015 at the onset of the flowering period, two inflorescences per individual in all treatments were randomly selected for measurement. Due to high flower production and their small size and fragility, three 10 cm sections were marked along each inflorescence (proximal, middle and distal), and we counted the number of flowers per racilla. From this, we extrapolated the number of flowers per inflorescence. Flowers were then left exposed to be naturally pollinated. Following fruit formation, the whole infructescences were covered with a mesh to estimate the total number of fruits produced by each individual. The following year (2015 and 2016, respectively), once the fruits were fully mature, they were collected. The probability of reproduction was estimated as the proportion of individuals which produced at least one fruit divided by total of individuals of each treatment.

#### Progeny performance

To assess the effect of defoliation on progeny performance, we conducted germination experiments using seeds produced in 2015 and 2016. The experiment was performed under controlled conditions in a greenhouse using a substrate with a 1:1 ratio of sand mixed with perlite and soil. Seeds were first placed in water to soften the fruit and remove the pericarp to facilitate the germination process. For 2015, we used the following numbers of seeds/mothers: N = 695/6 (C), N = 240/7 (L), and N = 106/6 (H); sample sizes for the 2016 cohort were: N = 558/6 (C), N = 98/7 (L) and N = 16/1 (H). The lower sample sizes were a result of steep declines in fruit production in the harvest treatments, particularly in 2016. For each year, we calculated the proportion of seeds germinated by treatment. We then measured growth (leaf production) of the resulting seedlings for nine months.

### Data analysis

To evaluate the cumulative effect of defoliation on *B*. *aculeata* over six years we analyzed eight different response variables classified in three groups: 1) leaf traits (leaf production, total leaves and leaf length), 2) reproductive success traits (flower production, fruit production and probability of reproduction) and 3) traits of progeny (germination and seedling growth). The explanatory variables were: i) Harvesting treatments (HT, with three levels: control, low harvest and high harvest) and ii) the time in years of study (Yr with six levels: 2011 to 2016) and the interaction between these two factors (H:T). These terms were considered as fixed factors, while the effect of repeated measures on individuals was considered as the random factor [37, 38]. For progeny traits (germination and seedling growth), the mother effect was included as a random factor [37]. Because the nature of response variables was different, the analyses were conducted using linear mixed models (LMM) for continuous variables (e.g. leaf length and seedling growth) and generalized linear mixed models (GLMM) for counts (e.g. leaf production, total leaves, flower and fruit production) and binomial variables (e.g. proportion of germination) [37–40], [42]. For continuous variables, such as leaf length and seedling growth, they were *log(x)* or *log (x + 1)* transformed to meet normality criteria. For count variables, a Poisson error and a logarithmic link function were used, while for binomial variables, a binomial error and a logistic link function were applied [38, 39]. All analyses were completed in R 3.3.3 [40]. LMM and GLMMs were conducted using the packages *lme4* ver 1.1–13 [38], [40], [42].

### Trade-offs

To investigate potential trade-offs between the vegetative and reproductive functions and if these become apparent when resources are reduced or depleted, we explored whether the relationship between leaf size and number of fruits changed over time (2010–2016). We used generalized linear mixed models (LMMs) to include the repeated measure effects on individuals as the random factor [37, 38]. As fixed factors, we included leaf size (LS) as the continuous variable and time of study in years (T) as the categorical variable. For each year, we obtained a model of the form *y = mx + b*, where *m* is the slope and *b* the ordinate. When significant, differences between slopes were tested among years. The regression lines presented in the results were obtained from these models. We conducted LMMs analysis using the *lme4* package of the R statistical software R 3.3.3 [41].

## Results

After six years of monitoring *Brahea aculeata* under different harvesting treatments, we found effects on all the analyzed traits. However, the leaf traits seem to be less affected by harvest than the reproductive ones.

### Leaf traits

**Fig 1:**
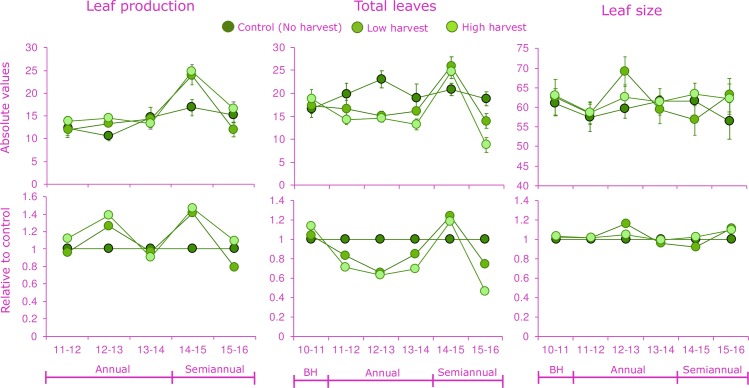
Variation in leaf traits of the *Brahea aculeata* under different experimental leaf harvest treatments in the tropical dry forest of Álamos, Sonora: Leaf production rate (leaves ind-1 yr-1), total leaves (leaves ind-1) and leaf length (cm). Top charts indicated the absolute values, while bottom charts indicated the proportional change compared to the control treatment for each year. At the bottom charts, the no harvest treatment (Control) is used as a reference.

Prior to the implementation of the harvest treatments (2010–2011), individuals from the three treatments had similar leaf production, total leaves and leaf size (Fig 1). In the case of leaf production, palms had a high variation with a mean (± SE) = 15.5 (± 0.6), but minimum and maximum of 2 and 35 leaves ind^-1^ yr^-1^, respectively. The statistical analysis indicated large differences among years, and significant interaction between harvesting treatment and time (H-T; Table 1). One year after the first annual harvesting (2012–2013) individuals from both low (L) and high (H) treatments showed an increase in new leaf production compared to control individuals (Fig 1). In 2014–2015, which was the first year with semiannual harvesting, control palms had the lowest leaf production rate, compared to individuals in the L and H treatments, that produced 1.5 and 1.6 times the number of leaves, respectively (Fig 1). However, for the final year of the experiment (2015–2016), leaf production decreased in the harvesting treatments especially for palms subjected to High harvest with 0.29 less leaf production (Fig 1 and Table 1).

**Table 1:** Results of the statistical analyses on leaf traits to evaluate the effects of chronic defoliation on *Brahea aculeata* in the tropical dry forest of Álamos, Sonora.

	Random effects		Fixed effects	
	Individuals	Harvesting (H)	Time (T)	H-T
	Variance	χ^2^ (df)	χ^2^ (df)	χ^2^ (df)
Leaf production	0.012	5.1^ns^(_2_)	124.6^***^(_4_)	17.1^*^(_8_)
Total number of leaves	0.013	11.9^**^(_2_)	131.6^***^(_5_)	50.9^***^(_10_)
Leaf length	0.007	1.1^ns^(_2_)	16.1^**^(_5_)	17.8^*^(_10_)

Mixed model statistics (χ^2^ and degrees of freedom) are provided. Significance level: *P < 0.05; **P < 0.01; ***P < 0.001; *ns*, non-significant. Terms tested were harvesting treatment (H), the time of harvesting in years (T) and the interaction between these factors (H-T). Repeated measurements on individuals through time was included in the model as a random factor (Individual) to account for the long-term design.

Statistical analysis indicated significant differences between harvest treatments (H), time (T) and the interaction of both factors (H-T, Table 1). The total number of leaves per individual of *B*. *aculeata* ranged from 8 to 26 (Fig 1). During annual frequency of harvesting, control palms had a higher number of total leaves that the harvested treatments. However, for the semiannual frequency period (2014–2016) as a result of higher leaf production, total number of leaves had the same pattern as the one above; that is, during 2014–2015 more leaves were observed in the defoliation treatments, and during 2015–2016, total number of leaves declined by 45% (L) and 35% (H). Statistical analysis indicated a significant interaction between harvest treatment and time (H-T; Fig 1 and Table 1). In addition to having the greatest leaf production rates, palms in the two harvest treatments (L and H) in general, also had longer leaves than control palms, especially at the 2012–2013 and 2015–2016 period (Fig 1). As a result of those differences, the mixed model identified differences among years and a significant interaction H-T; Table 1).

### Reproductive success

**Fig 2:**
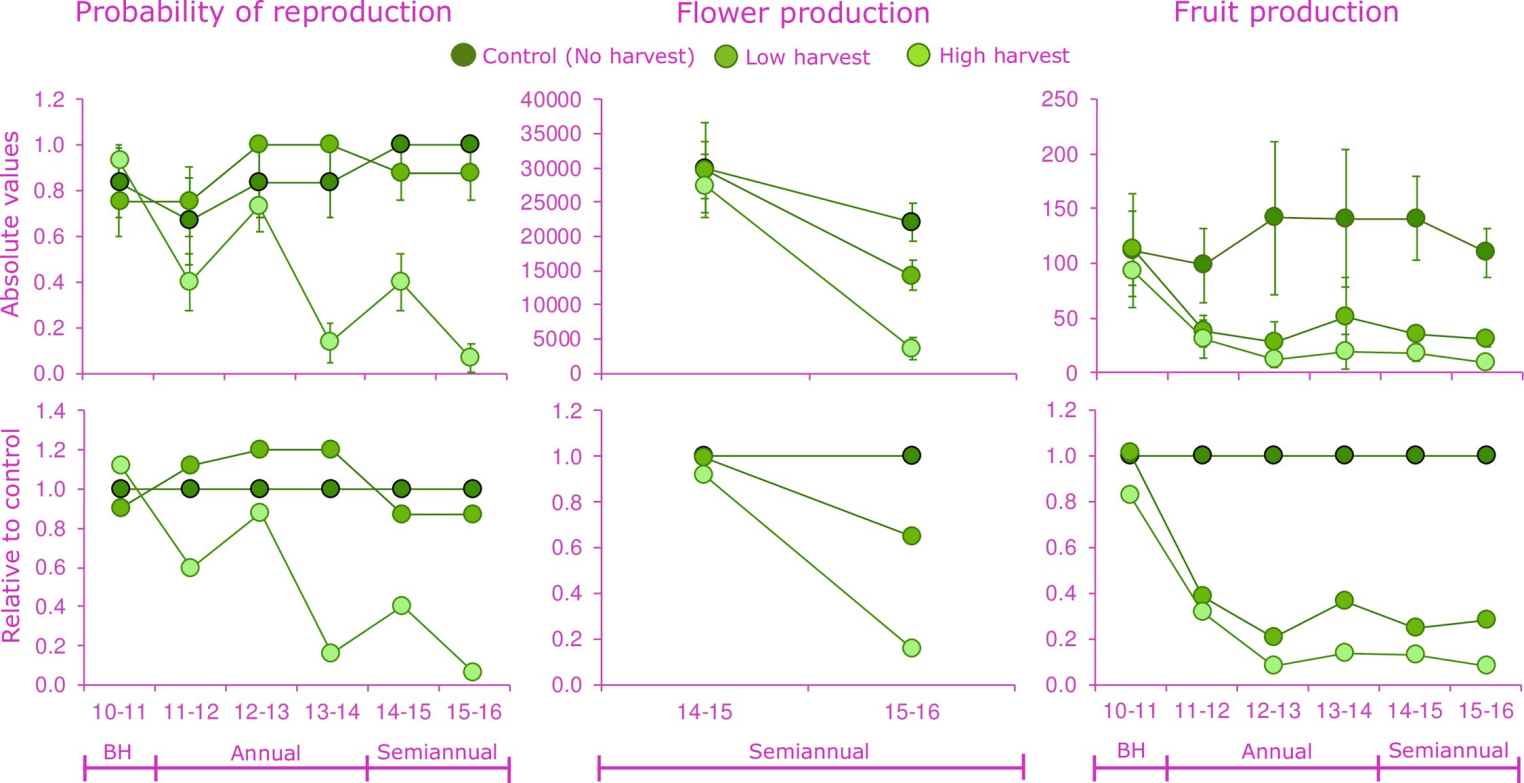
Variation in reproductive traits of *Brahea aculeata* under different experimental leaf harvest treatments in the tropical dry forest of Álamos, Sonora: Probability of reproduction (ind ind-1 yr-1), flower production (flowers ind-1 yr-1) and fruit production (fruits ind-1 yr-1). Flowers were only during the two last years. Top charts indicated the absolute values, while bottom charts indicated the proportion of change compared to the control treatment for each year. At the bottom charts, the no harvest treatment (Control) is used as a reference.

The pre-treatment (2010–2011) probability of reproduction and fruit production were similar among the three treatments, but the harvesting treatment showed significant reduction in all the analyzed reproductive traits (Fig 2). The probability of reproduction was a very sensitive trait, especially for palms subjected to high harvest. These individuals had dramatically reduced probability of reproduction from the first harvest and dropped up to 0.18 in 2013–2014 period (Fig 2). During the semiannual harvest, probability of reproduction of high-harvested palms kept dropping and only one out of 15 individuals reproduced (probability 0.06).

In 2014–2015, similar flower production was recorded in both harvest treatments (L and H) than in control palms (Fig 2). However, one year after (2015–2016) a reduction was observed in all treatments including controls, though this last continued to have a higher average number of flowers than individuals in both harvest treatments, which make the mixed model found the harvesting treatments (H) and the interaction (H-T) to be significant (Fig 2 and Table 2). Fruit production had a big variation over time, especially in control individuals, though it was very evident that individuals in the harvest treatments produced significantly fewer fruits reducing dramatically since first annual harvest (Fig 2). By the end of the experiment, individuals subjected to harvesting had the lowest fruit production with 0.73 and 0.91 reduction compared to control palms (Fig 2). Variation in fruit production among treatments by year resulted in a significant interaction (H-T; Table 1).

**Table 2:** Results of the statistical analyses on reproductive traits to evaluate the effects of chronic defoliation on *Brahea aculeata* in the tropical dry forest of Álamos, Sonora.

	Random effects	Fixed effects		
	Individual	Harvesting (H)	Time (T)	H:T
	Variance	χ^2^ (df)	χ^2^ (df)	χ^2^ (df)
Probability of reproduction	0.23	16.4**(2)	17.1*(5)	13.4^ns^_(10)_
Flowers	0.12	7.9^*^_(2)_	154.9^***^_(5)_	90.7^***^_(10)_
Fruits	3.17	12.9^**^_(2)_	168.1^***^_(5)_	64.3^**^_(10)_

Mixed model statistics (χ^2^ and degrees of freedom) are provided. Significance level: *P < 0.05; **P < 0.01; ***P < 0.001; *ns*, non-significant. Terms tested were harvesting treatment (H), the time of harvesting in years (T) and the interaction between these factors (H-T). Repeated measurements on individuals through time was included in the model as a random factor (Individual) to account for the long-term design.

### Progeny performance

**Fig 3:**
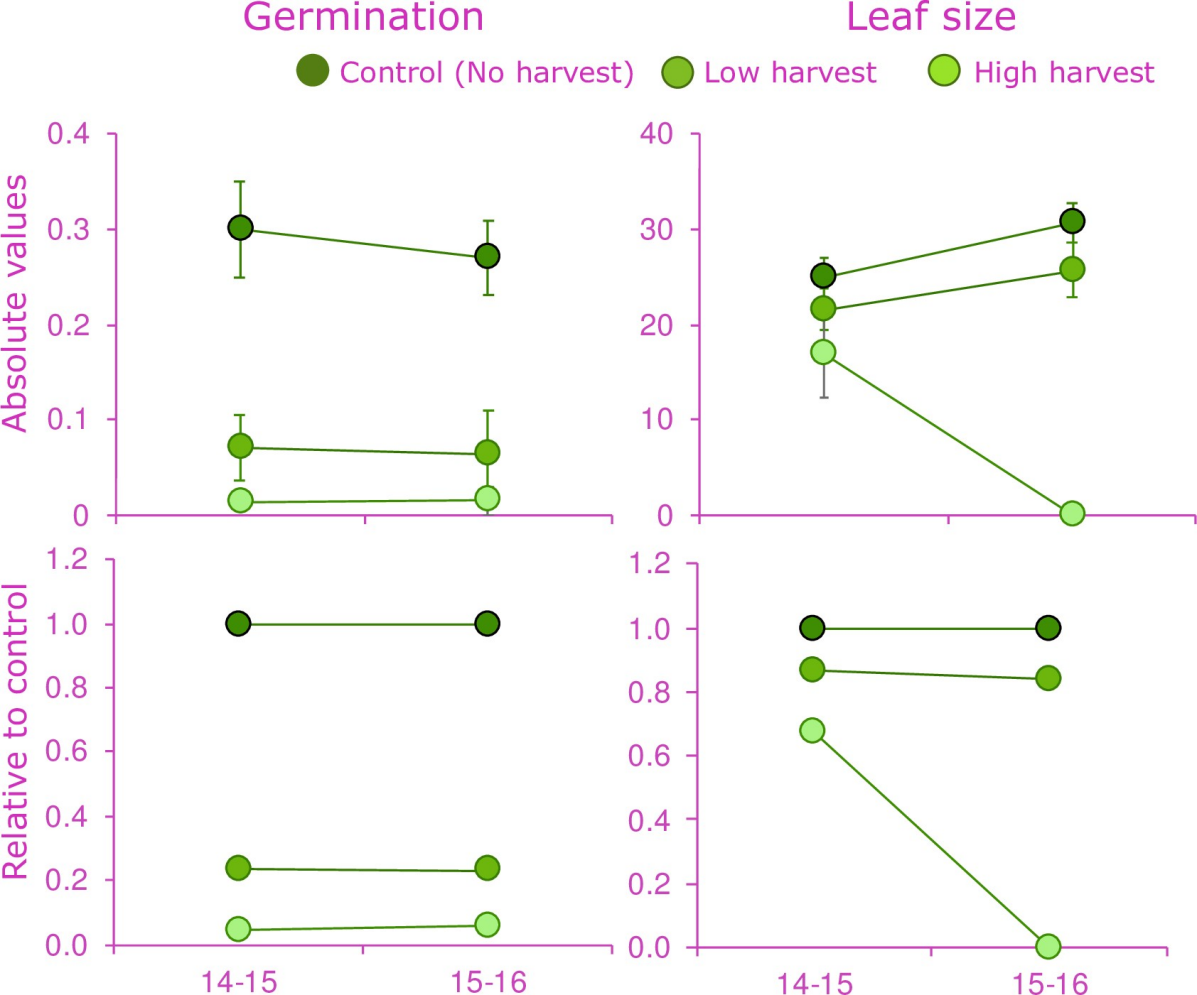
Variation in the performance of the progeny of *Brahea aculeata* under different experimental treatments of leaf harvesting in the dry forest of Álamos, Sonora: Germination rate (germinated seeds mother-1 yr-1), b) Leaf length of seedlings (cm). Top charts indicated the absolute values, while bottom charts indicated the proportional change compared to the control treatment for each year. At the bottom charts, the no harvest treatment (Control) is used as a reference.

Germination and subsequent seedling growth, as indicated by the leaf size at the 9^th^ month of monitoring, differed significantly between the harvest treatments and the control treatment (Table 3), with reduced germination and slower growth of seedlings in the harvest treatments. Germination rates in the low and high harvesting were below 5%. While, germination rates from seeds obtained from the control treatment were also low (0.31), the rate was significantly higher than both treatments (Fig 3 and Table 3). Significant differences in seedling growth among treatments (H) and time (T) were also found (Table 3), with highest mean leaf size in the control treatment during the 9 months of observation (Fig 3).

**Table 3:** Results of the statistical analyses on progeny traits to evaluate the effects of chronic defoliation on *Brahea aculeata* in the tropical dry forest of Álamos, Sonora.

	Random effects	Fixed effects		
	Variance	Harvesting (H)	Time (T)	H:T
	Mother	χ^2^(df)	χ^2^(df)	χ^2^(df)
Germination	0.14	36.4^***^_(2)_	0.5^ns^_(1)_	2.4^ns^_(2)_
Leaf size	14.9	72.5^***^_(2)_	4.4*_(1)_	56.4***_(2)_

Mixed model statistics (χ^2^ and degrees of freedom) are provided. Significance level: *P < 0.05; **P < 0.01; ***P < 0.001; ns, non-significant. The table presents only significant terms. Terms tested were harvesting treatment (H), the time of harvesting in years (T) and the interaction between these factors (H-T). The mother effect were included in the model as random factor.

### Trade-offs between foliar and reproductive traits

**Fig 4:**
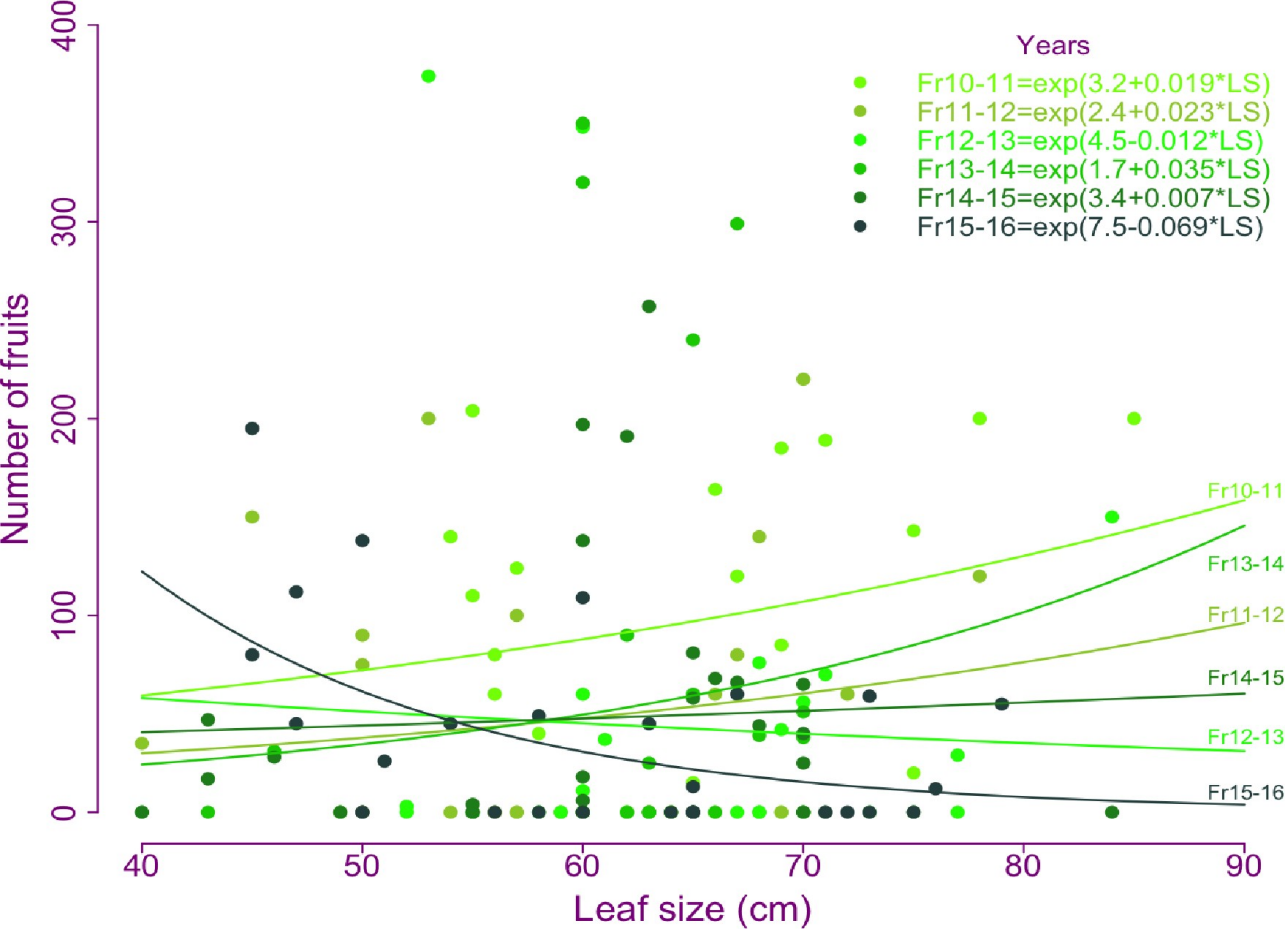
Trade-off between the leaf size and fruit production of *Brahea aculeata* under different experimental treatments of leaf harvesting in the dry forest of Álamos, Sonora. The figure shows the coefficients (intercept and slope) indicated by the model for each year. Symbols represent the different years, respectively. The lines in color gradient represents the prediction of the model for each year and are indicated next to the line.

The GLMM indicated that fruit production was independent of leaf size (LS: χ^2^_1_ = 1.8, p = 0.17). However, we found that fruit production changed among years (T: χ^2^_5_ = 122.6, p<0.001*) with higher fruit production for the 2010–2011 period, which correspond to the year previous to harvest. The relationship between fruit production and leaf size, changed gradually through the time of the study which resulted in a significant interaction (LS-T: χ^2^_5_ = 109.9 p<0.001*). For the first 5 (2010–2015) periods of study, the GLMM indicated a positive relationship, with the highest slope found for 2013–2014 period (*m = -0*.*035 fruits/cm*) and for 2014–2015, the model indicated a slope nearly zero (*m = -0*.*007 fruits cm*^*-1*^; Fig 4). However, for the last period (2015–2016), which was the second one with semiannual harvesting, the slope becomes negative (*m = -0*.*069 fruits cm*^*-1*^) indicating lower fruit production for individuals that had produced larger leaves (Fig 4).

## Discussion

The results from our study indicate that defoliation of reproductive *Brahea aculeata* individuals alters resource allocation patterns of essential functions such as growth, maintenance and reproduction [43, 44]. In general, palms in both harvested treatments initially showed increases in leaf production and leaf length, a similar response to what has been reported in most of the studies focused on evaluating the effects of defoliation on palms and has been proven or interpreted as overcompensation [8], [10], [30], [13, 14], [45, 46]. However, this pattern changed the following year where we observed slight declines, especially in the number of leaves, but also for leaf length and production. Thus, the foliar traits of *B*. *aculeata* reproductive individuals after five years of defoliation showed resilience and continue to maintain an over-compensatory response [21], [30]. Possibly, the mechanism by which these individuals mitigate leaf area loss is by allocating more resources to vegetative maintenance and growth functions [8], [43]. In this case, plants could be recovering the leaf area as a tolerance strategy, since these leaves are essential for energy uptake and plant functions [47, 48]. This behavior observed *in B*. *aculeata* is similar to other studies, where intensely harvested individuals allocated more resources to vegetative structures and growth and less to reproductive structures [14]. In addition, if this harvest continues chronically, the negative effects on reproduction may be long, taking years to recover; as has been found for other palms [8], [10], [19], [49]. Additionally, because reproduction has a higher cost compared to vegetative costs, it is also expected to cause negative consequences on progeny [21], [50]. Thus, the chronic leaf area loss will affect firstly the reproductive function, followed by growth and if leaf area loss continues, the risk of mortality of individuals increases [8], [51–53].

### Progeny performance

Our results also demonstrate the negative effects of defoliation on the progeny, specifically on the viability of seeds and seedlings growth. For palms, defoliation impacts on progeny, is a novel finding with large implications for long-term sustainability of species used such as NTFPs [4], [28], [52]. This has also been shown in other species under natural herbivory; such as *Piper arieianum* and *Psychotria horizontalis* [54, 55]. This is not a general pattern for plants, as it has also been reported no-effects or positive effects on the production of seeds [56, 57]. The results found in the offspring are highly relevant, as it appears that the resources in the plant are depleted and are assigned in less proportion to the reproductive function [58]. Thus, the effects of leaf area loss may be also trans-generational, becoming evident in mother plants that suffer from defoliation and in their descendants [59]. Apparently, high and sustained defoliation in *B*. *aculeata*, as in other species of palms, deplete the resources available in the plant, affecting first the reproductive function, and if the harvest continues there will be a reduction in growth and finally survival [8], [53].

To our knowledge most of the studies on leaf-harvesting have explored the effects from the physiological, phenological and/or morphological view [13, 14], [46], [49]. Also, many have determined the effects on vegetative and reproductive traits, or the strategies of resource allocation between maintenance/growth/reproduction traits [7], [27], [60]. However, with very few exceptions, most of these studies have conducted experiments only within a single generation and have not evaluated the transgenerational effects [58]. Our finding of reduced germination and growth of progeny in response to adult leaf harvest is an issue that has, to date, rarely been considered when assessing the ecological effects of NTFP harvest or the sustainability of extraction [27]. These results indicate that even in cases where other demographic attributes such as survival or growth are suggestive of little or no effect of harvest on vital rates, effects on the fitness of progeny may be substantial, resulting in population declines in a long-term approach. Additional research is needed to better understand if this is a broader trend within palms or NTFP species, or restricted to species such as *B*. *aculeata*.

### Trade-offs between foliar and reproductive traits

According to the results obtained in vegetative and reproductive traits in the last year of defoliation, the cumulative effect of the harvest on *B*. *aculeata* was evident, observing trade-offs between these traits. It has been demonstrated that those compromises are resource and condition dependent [55], [61]. A clear example is observed in *Sagittaria pygmaea*, where there are no trade-offs between sexual and asexual reproduction at high concentrations of nutrients, but when the amount of nutrients is low or moderate they become clearly significant [62]. This pattern can be explained by the principle of resource allocation, which means that when two functions had a negative relationship, the higher investment of resources into a single function causes decreases in energy assigned to other functions essential to the plant including defenses [20], [50], [55]. This re-assignation involves a complex trade-off between vegetative and reproductive traits. We believe for non-timber forest products this is a novel finding, which needs to be further studied to be able to understand the transgenerational effects that leaf area loss may have [21]. This management, which includes chronic defoliation, may be shaping the population structure and long-term dynamics, but also the genetic variability of the population.

### Implications for management and conservation

We acknowledge that at the end of our experiment we had low sample sizes for some treatments. However, manipulative experiments focused on leaf harvest going beyond 2–3 years are rare, and our findings, particularly with respect to the effects on progeny performance expand our understanding of how leaf harvest affects vital rates, resource allocation, and population dynamics. We believe that our results are consistent and can be very useful to inform on sustainable harvesting of our study species and other similar NTFP.

Several studies have highlighted that long-term studies focused on multiple ecological levels are necessary for species used as NTFP, especially for those harvested recurrently used [4], [18], [63]. However, only few have accomplished this with important novelties that help to understand the population dynamics in real scenarios [8]. Short-term studies may not capture the actual effects of chronic harvesting and inaccurate conclusions can be drawn [4]. Through long-term studies, the different mechanisms allowing plants to recover from intensive and frequent harvest can be explored. Results from the first three years of our study did not allow us to detect the negative effects of harvesting on reproduction [30]. Our recent findings indicated strong negative effects on the resource allocation patterns and especially to those allocated to reproduction and progeny. To our knowledge among leaf-harvesting studies, this transgenerational effect has not been evaluated. We believe this finding is a novel aspect to consider in further studies of leaf-harvesting aimed to generate useful information for long-term sustainable management of NTFP, such as palms, which represent some of the most important species. Also, it would be interesting to explore if plants and progeny traits are able to recover to the pre-defoliation stage and how long this recovery process may require [8].

The abundance of *Brahea aculeata* in northern Mexico has diminished and we believe our results can be useful to explain how the chronic and intensive harvest practices by some groups of people in the area may have contributed to the species’ reduction [29]. Also, seeds of *Brahea aculeata* represent an important resource for an important group of frugivorous mammals [64] and therefore intensive harvesting schemes may also affect other components of the ecosystem. Results from this research may contribute to design sustainable harvesting programs considering intensity, frequencies and periods for recovery from defoliation. Also, our findings may help to explain how intensive and non-planned management schemes may negatively affect vital rates and long-term dynamics of populations from non-timber forest products and other components of the ecosystem [18]. Finally, our results, may also be important for other non-timber forest products, especially from dry forests, where little or no information exists.
